# Assessment of PEEP-Ventilation and the Time Point of Parallel-Conductance Determination for Pressure-Volume Analysis Under β-Adrenergic Stimulation in Mice

**DOI:** 10.3389/fcvm.2019.00036

**Published:** 2019-04-10

**Authors:** Lucas Bacmeister, Sebastian Segin, Rebekka Medert, Diana Lindner, Marc Freichel, Juan E. Camacho Londoño

**Affiliations:** ^1^Pharmakologisches Institut, Ruprecht-Karls-Universität Heidelberg, Heidelberg, Germany; ^2^Partner Site Heidelberg/Mannheim, DZHK (German Centre for Cardiovascular Research), Heidelberg, Germany; ^3^Allgemeine und Interventionelle Kardiologie, Universitäres Herzzentrum Hamburg, Hamburg, Germany; ^4^Partner Site Hamburg/Kiel/Lübeck, DZHK (German Centre for Cardiovascular Research), Hamburg, Germany

**Keywords:** pressure-volume analysis, parallel-conductance, β-adrenergic stimulation, hypertonic saline, positive end-expiratory pressure, end-systolic pressure-spikes, catheter entrapment, hemodynamics

## Abstract

**Aim:** Cardiac pressure-volume (PV loop) analysis under β-adrenergic stimulation is a powerful method to simultaneously determine intrinsic cardiac function and β-adrenergic reserve in mouse models. Despite its wide use, several key approaches of this method, which can affect murine cardiac function tremendously, have not been experimentally investigated until now. In this study, we investigate the impact of three lines of action during the complex procedure of PV loop analysis: (i) the ventilation with positive end-expiratory pressure, (ii) the time point of injecting hypertonic saline to estimate parallel-conductance, and (iii) the implications of end-systolic pressure-spikes that may arise under β-adrenergic stimulation.

**Methods and Results:** We performed pressure-volume analysis during β-adrenergic stimulation in an open-chest protocol under Isoflurane/Buprenorphine anesthesia. Our analysis showed that (i) ventilation with 2 cmH_2_O positive end-expiratory pressure prevented exacerbation of peak inspiratory pressures subsequently protecting mice from macroscopic pulmonary bleedings. (ii) Estimations of parallel-conductance by injecting hypertonic saline prior to pressure-volume recordings induced dilated chamber dimensions as depicted by elevation of end-systolic volume (+113%), end-diastolic volume (+40%), and end-diastolic pressure (+46%). Further, using this experimental approach, the preload-independent contractility (PRSW) was significantly impaired under basal conditions (−17%) and under catecholaminergic stimulation (−14% at 8.25 ng/min Isoprenaline), the β-adrenergic reserve was alleviated, and the incidence of ectopic beats was increased >5-fold. (iii) End-systolic pressure-spikes were observed in 26% of pressure-volume recordings under stimulation with 2.475 and 8.25 ng/min Isoprenaline, which affected the analysis of maximum pressure (+11.5%), end-diastolic volume (−8%), stroke volume (−10%), and cardiac output (−11%).

**Conclusions:** Our results (i) demonstrate the advantages of positive end-expiratory pressure ventilation in open-chest instrumented mice, (ii) underline the perils of injecting hypertonic saline prior to pressure-volume recordings to calibrate for parallel-conductance and (iii) emphasize the necessity to be aware of the consequences of end-systolic pressure-spikes during β-adrenergic stimulation.

## Introduction

Cardiac pressure-volume (PV) analysis during incremental β-adrenergic stimulation in mice is capable of precisely determining ventricular function and adrenergic reserve in mice. This species offers advantages like the feasibility of genetic modifications and short generation times. Established since 1998 (1), PV analysis in mice revealed numerous novel regulators of cardiac function and is still regarded as the gold standard characterizing ventricular function (2, 3).

PV analysis is performed by advancing a pressure-conductance catheter into the left ventricle. A piezo-electrical pressure transducer continuously detects intra-ventricular pressure. Ventricular volume is measured by generating an electrical field using the catheter's two outer electrodes. Conductance-alterations within the blood-filled chamber are detected by two inner electrodes. Assuming a cylindrically shaped left ventricle, conductance can be converted into volume (4). However, the measured overall conductance (G) reflects of the blood-conductance within the ventricle (G_b_) and the conductance of surrounding tissue (parallel-conductance, G_p_). To obtain G_b_, G_p_ needs to be subtracted from G. G_p_ may be estimated by injecting hypertonic saline into the left ventricle and by subsequent analysis of PV recordings during the wash-in phase as first described in canine (4, 5) and later validated for mice (6–8).

Several PV protocols assessing murine cardiac function have been published (1, 2, 9, 10). Moreover, the influence of several steps during the procedure, such as approaches using open-chest or closed-chest surgery (9, 11), the choice of anesthetics (11) as well as the genetic background (12) have already been examined regarding their impact on cardiac function. However, other factors potentially affecting basal cardiac function and adrenergic reserve have not been investigated until now. In this study, we performed PV analysis during incremental Isoprenaline stimulation following open-chest access to the heart and evaluated three key methodological aspects during the procedure of PV loop analysis in mice. We (i) ventilated mice with or without positive-end-expiratory pressure (PEEP), (ii) we estimated G_p_ by injecting hypertonic saline either prior to or after PV recordings, and (iii) we analyzed which PV parameters are affected upon appearance of end-systolic pressure-spikes (ESPS), a common phenomenon observed in PV analysis under adrenergic stimulation (1, 8, 13–15).

Our findings (i) demonstrate the advantages of PEEP ventilation under β-adrenergic stimulation in open-chest instrumented mice, (ii) reveal multiple adverse effects of estimating G_p_ by injecting hypertonic saline prior to PV recordings, and (iii) show ESPS occurring under β-adrenergic stimulation do affect both pressure- and volume-related parameters in the analysis of cardiac function.

## Materials and Methods

### Animals

All experimental procedures fulfilled the EU-legislation guidelines for protection of animals used for scientific purposes and were approved by the Karlsruhe regional council (G131/15 and G121/17). Recordings from 48 male C57Bl/6N mice aged 19.4 ± 1.4 weeks were analyzed. Animals were obtained from Charles River Laboratories and housed in a 12 h light cycle under specified pathogen-free conditions (SPF) at the animal facility (IBF) of the Heidelberg Medical Faculty. Rodent food (LasVendi Rod18) and drinking water were supplied *ad libitum*.

### Anesthesia

Anesthesia was induced in a plexiglas-chamber pre-saturated with 2.5% Isoflurane, vaporized with 80 ml/min O_2_ (Vapor 19.3, Abbot). Subsequently an anesthetic-mixture (7 ml/kg) containing 0.1 mg/kg Buprenorphine as analgesic (Bayer AG), 1,200 IU/kg Heparin (Ratiopharm GmbH) and 10 mg/kg Etomidate-Lipuro (B. Braun Melsungen AG) was injected intraperitoneally (i.p.). Anesthetized animals were endotracheally intubated using the shortened (3 cm) plastic cannula of an intravenous 20 Gauge catheter. After fixation on a heating plate (Hotplate 062, Labotect) a rectal probe (Hugo Sachs Elektronik) was inserted. Body temperature was maintained at 37 ± 0.5°C by manually adjusting heating plate temperature to 38.5 ± 1.5°C. A small rodent respirator (MiniVent 845, Harvard Apparatus) was, connected to the endotracheal tube. On-line airway pressures were monitored in LabChart 7.3 Pro (AD Instruments) by connecting a MPX-Transducer (Hugo Sachs Elektronik) to the respiratory tube. Ventilation was performed by setting respiratory rates to 53.5 × M^0.26^ [min^−1^] (M = BW in g) (9) and adjusting tidal volumes to peak inspiratory pressures of 11 ± 1 cmH_2_O. After set once at the beginning, tidal volume was not altered during experiments. Adequate anesthesia depth was proved with a tail pinch and the absence of reflexes at the hind paws. Hereafter, 1 mg/kg Pancuronium (Sigma Life Science) was injected i.p. to prevent respiratory artifacts during recordings. The range of Heart Rate as an indicator for anesthesia depth (16) was monitored on-line in LabChart 7.3 Pro with a subcutaneous 1-lead ECG (Bio Amp, AD Instruments).

### Surgery

During surgery, anesthesia was maintained by ventilating with 2% Isoflurane vaporized with 80 ml/min O_2_. All surgical procedures were performed under magnification (1.5- to 4-fold) via a surgical microscope (Stemi 2000-C, Carl Zeiss). Access to the central venous system was established by cannulation of the left femoral vein (LFV, Figure S1A).

In all experimental groups, fluid-loss was counteracted by infusing 15 μl/min 0.9% sodium-chloride (NaCl) with a syringe pump (11Plus, Harvard Apparatus). Hereafter, a thoracotomy, as adapted and modified from others (2, 9), was performed (Figure S1B). Two cmH_2_O positive end-expiratory pressure (PEEP) was established in 38 of 48 mice just before diaphragm incision by placing the expiratory ventilation hose 2 cm beneath the water-surface. To validate the established PEEP, on-line airway-pressures were monitored in LabChart 7.3 Pro. After diaphragm incision, a limited costotomy was performed (Figure S1B). The pericardium was bluntly dissected and a suture (8.0, Suprama) was positioned beneath the inferior caval vein (ICV). Finally, the left ventricular apex was pricked with a 25 gauge needle, and a 1.4-F pressure-conductance catheter (SPR-839, Millar) was inserted (Figure S1C). Intra-ventricular catheter position was optimized under on-line visualization until rectangular-shaped loops were obtained.

### Pressure-Volume Measurements

Isoflurane was reduced to 1.5% and animals were allowed to reach steady state conditions as indicated by constant heart rate and stroke volume, which was observed within 3–5 min. If the stabilization period extended 10 min, animals were not included into the analysis. The first two cycles of recording were performed while infusing 15 μl/min 0.9% NaCl. PV loops were recorded during two cycles of inferior caval vein (ICV) occlusion for each Isoprenaline (ISO) dose, after a new steady state was reached. To maintain constant infusion rates of 15 μl/min, increasing doses of 0.2475, 0.825, 2.475 and 8.25 ng/min Isoprenaline (ISO, Sigma Life Science) dissolved in 0.9% NaCl were infused by switching syringes. As the respiratory status has a significant impact on murine cardiac function during PV recordings (Figure S2), ventilation was suspended strictly at the end-expiration time point. Preload dependent parameters were recorded during the first 2 s of a measurement (Figure S3). To obtain preload-independent parameters, the suture around the ICV was gently lifted for the following 2 s of each measurement (Figure S3).

### Calibration

To estimate G_p_, 10 μl hypertonic saline (15% NaCl) were rapidly injected into the left femoral vein (4, 5, 8). In 33 animals, this was performed after measurements (late saline group), whereas 15 mice received hypertonic saline prior to measurements (early saline group). Each experiment was completed by sampling left ventricular blood 5 min after saline-injection in the late saline group, or directly after the last measurement in the early saline group. Subsequently, mice were euthanized by massive bleeding after removal of the heart under anesthesia and Buprenorphin analgesia. Hereafter, blood conductance was measured in pre-warmed (37°C) cylinders of known volume (Millar).

### Pressure-Volume Analysis

All analytical steps were performed in LabChart 7.3 Pro. A factor for conductance-to-volume conversion (conversion-factor, CF) was enabled for each dataset by calibrating the known volumes of the calibration cuvette against blood-conductance. Subsequently, PV recordings during hypertonic saline's left ventricular wash-in phase were analyzed. G_p_ was then subtracted from G.

For baseline analysis, 5–10 consecutive loops during end-expirational ventilation pause were selected (Figure S3). Preload-independent parameters were analyzed by selecting loops during ICV-occlusion (Figure S3). To ensure standardized calculation of preload independent parameters, solely the first five consecutive loops showing decreasing V_ed_ were analyzed (Figure S3).

Parameters used to characterize general cardiac function were heart rate (HR), stroke work (SW), stroke volume (SV), cardiac output (CO), maximum pressure (P_max_), end-diastolic pressure (P_ed_), end-diastolic volume (V_ed_) and end-systolic volume (V_es_). Preload independent cardiac contractility was analyzed by Preload Recruitable Stroke Work (PRSW) (17). Preload dependent cardiac contractility was analyzed by maximum dP/dt (dP/dt_max_). Isovolumetric relaxation was analyzed by Tau (Weiss Equation) (18) and minimum dP/dt (dP/dt_min_). Diastolic filling was analyzed by End-diastolic Pressure-Volume Relationship (EDPVR, alpha-coefficient).

### Analysis of Airway Pressures

Peak inspirational airway pressures (Peak P_insp_), were first determined when the conductance-catheter was inserted into the ventricle (t_0_) and followed up 5, 10, and 15 min after catheter insertion (t_5_, t_10_, and t_15_). Five consecutive Peak P_insp_ were averaged for each time point.

### Analysis of Ectopic Beats

Ectopic beats were analyzed by dividing each experiment into sections of 1 min intervals, starting after steady state conditions were reached until the last ICV occlusion. If ectopic beats occurred during a section, this section was considered as “section with ectopic beat.” Ectopic beats occurring timely associated with ICV occlusions were excluded from analysis, as these could have been ectopically driven by a contact between occlusion-suture and cardiac structures. Figure S4 depicts how ectopic beats were detected in the PV analysis and in the simultaneous ECG.

### Statistical Analysis

Statistical analysis and figures were done in OriginPro 2016 and Excel. Two-sided, unpaired Student's *t*-tests were performed, and heteroskedastic variances were assumed (Microsoft Excel 2011). For analysis of increases of Peak P_insp_ within groups two-sided, paired Student's *t*-tests assuming heteroskedastic variances were performed (Microsoft Excel 2011). *P*-values < 0.05 were considered statistically significant. All data is presented as mean ± standard deviation.

## Results

### The Effect of Positive End-Expiratory Pressure (PEEP) Ventilation

**Figure 1:**
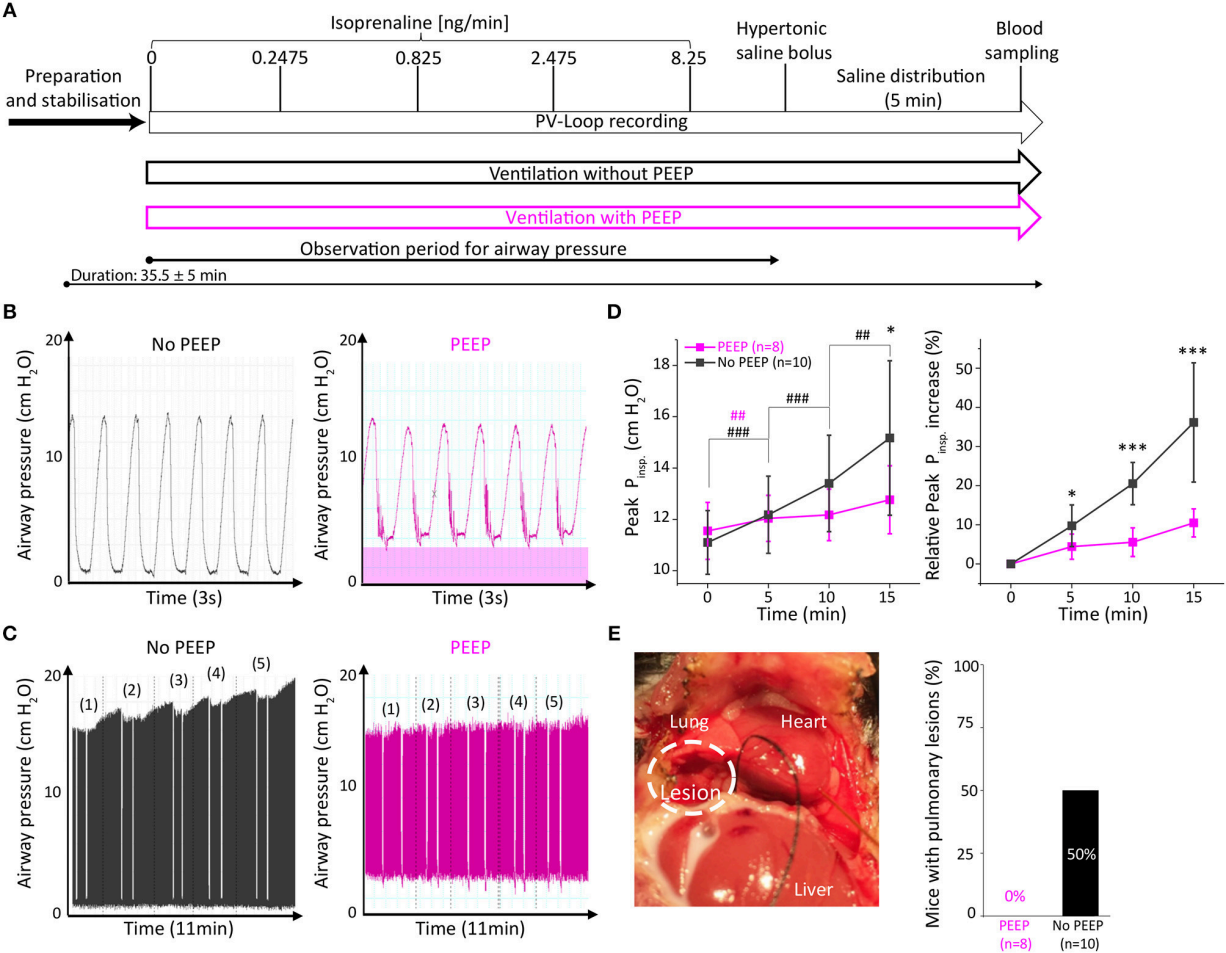
Ventilation with positive end-expiratory pressure (PEEP) protected mice from exacerbated airway pressure and prevented pulmonary lesions. **(A)** Experimental design with two groups either ventilated without PEEP (black) or with PEEP of 2 cmH_2_O (purple). Comparison of representative traces of short-term **(B)** and long-term **(C)** PEEP-ventilation. In **(C)** white incisions depict end-expirational ventilation-stops during PV loop recordings. **(D)** Analysis of peak inspiratory pressure (Peak P_insp_) during 15 min after catheter insertion (time 0). **(E)** Representative picture of a pulmonary lesion which occurred in 5 out of 10 non-PEEP-ventilated animals and quantitative assessment of pulmonary lesions. Data presented as mean ± standard deviation from *n* = 10 (No PEEP group) and *n* = 8 (PEEP group) mice. *n*, number of mice. *p < 0.05, ***p < 0.001: *p*-value No PEEP vs. PEEP in an unpaired Student's *t*-test; #*p* < 0.05, ##*p* < 0.01, ###*p* < 0.001: *p*-value vs. previous corresponding observation point in a paired Student's *t*-test within the groups (purple #: PEEP group, black #: No PEEP group).

To analyse the effect of positive end-expiratory airway pressure (PEEP) on open-chest PV loop recordings under β-adrenergic stimulation, we measured two groups of adult C57Bl6/N mice. The experimental protocol in both groups did not differ except for the ventilation, which was performed either with or without PEEP (2 cmH_2_O, Figure 1A). The course of events after intubation including ventilation, venous catheterisation, incremental β-adrenergic stimulation and hypertonic saline injection is indicated in Figure 1A.

As PEEP-ventilation may alter airway pressures during open-chest PV loop analysis, we examined peak inspirational pressures (Peak P_insp_) immediately after catheter insertion and 5, 10, and 15 min thereafter. Representative traces indicate the increase in end-expiratory airway pressures in PEEP-ventilated animals (Figure 1B). Interestingly, a time-dependent increase of Peak P_insp_ was observed in animals that were not ventilated with PEEP (Figures 1C,D). Following 15 min of PV loop recording, Peak P_insp_ increased by 36.2 ± 15.2% in the non-PEEP ventilated group as compared to 10.5 ± 3.6% in the PEEP-ventilated group (*p* = 0.0004, Figure 1D). Notably, macroscopic pulmonary bleedings were exclusively observed in 5 out of 10 (50%) non-PEEP-ventilated animals but in none of the PEEP-ventilated animals (Figure 1E), suggesting that pulmonary lesions develop along with exacerbated Peak P_insp_. In contrast, body temperature, calibration factors and hypertrophy indices did not differ between the two groups (Table S1).

Since a stop in end-inspirational ventilation may affect cardiac function due to increased lung volume and airway pressure in PV analysis (Figure S2), we examined the effect of 2 cmH_2_O positive airway pressure in PEEP ventilated animals on PV loop parameters in comparison to the non-PEEP ventilated group. PEEP ventilation did not affect basal systolic, diastolic cardiac performance, or β-adrenergic reserve (Table 1).

**Table 1:** Pressure-volume analysis under β-adrenergic stimulation from mice with or without positive end-expiratory pressure ventilation.

	**Systolic parameters**			**Diastolic parameters**		
**Isoprenaline (ng/min)**	**No PEEP (*n* = 10)**	**PEEP (*n* = 8)**	***p*-Value**	**No PEEP (*n* = 10)**	**PEEP (*n* = 8)**	***p*-Value**
	**Heart rate (bpm** **±** **SD)**			**End-diastolic volume (μl** **±** **SD)**		
0	498 ± 35	494 ± 33	0.7918	24.4 ± 5.8	24.9 ± 3.6	0.8034
0.2475	518 ± 34	514 ± 33	0.8115	23.6 ± 6,1	23.7 ± 3.6	0.9511
0.825	554 ± 26	546 ± 22	0.4704	21.4 ± 4.4	22.3 ± 3.2	0.6220
2.475	597 ± 22	612 ± 25	0.2008	21.4 ± 3.2	21 ± 3.2	0.7856
8.25	620 ± 20	620 ± 20	0.9985	22.7 ± 3.4	23.2 ± 2.6	0.7028
	**Stroke volume (μl** **±** **SD)**			**End-systolic volume (μl** **±** **SD)**		
0	17.9 ± 2.9	18.9 ± 2.8	0.4707	7.78 ± 6.97	7.39 ± 4.03	0.8839
0.2475	18.7 ± 2.5	19.6 ± 2.5	0.4453	6.22 ± 7.56	5.33 ± 3.63	0.7459
0.825	19.9 ± 2.9	20.4 ± 2.5	0.6895	2.86 ± 3.7	2.94 ± 2.27	0.9515
2.475	21.9 ± 2.5	22.3 ± 1.9	0.6946	1.12 ± 1.18	0.43 ± 0.33	0.1023
8.25	23.5 ± 2.3	24.3 ± 1.8	0.4394	1.15 ± 1.04	0.4 ± 0.31	0.0532
	**Cardiac output (μl/min** **±** **SD)**			**End-diastolic pressure (mmHg** **±** **SD)**		
0	8,872 ± 1,388	9,329 ± 1,600	0.5343	5.07 ± 1.46	5.52 ± 1.26	0.4920
0.2475	9,717 ± 1,558	10,115 ± 1,574	0.5993	4.94 ± 1.61	5.26 ± 1,12	0.6199
0.825	11,016 ± 1,755	11,141 ± 1,624	0.8780	4.95 ± 1.67	5.5 ± 0.93	0.3933
2.475	13,089 ± 1,648	13,646 ± 821	0.3668	4.44 ± 1.28	5.4 ± 0.84	0.0738
8.25	14,559 ± 1,311	15,046 ± 1,151	0.4136	4.65 ± 1.16	5.47 ± 0.78	0.0924
	**Maximum pressure (mmHg** **±** **SD)**			**EDPVR (α** **±** **SD)**		
0	82.1 ± 6	85.5 ± 5.7	0.2389	1.68 ± 1.38	1.64 ± 1.04	0.9419
0.2475	84.5 ± 6.8	88.3 ± 8.1	0.3093	1.66 ± 1.7	1.54 ± 0.9	0.8461
0.825	87.7 ± 5.9	87.5 ± 8.1	0.9545	1.69 ± 1.47	1.88 ± 0.71	0.7258
2.475	89.2 ± 6.3	90.3 ± 7.5	0.7589	1.52 ± 0.91	2.41 ± 0.58	**0.0249**
8.25	90.2 ± 6	91.2 ± 5.8	0.7289	1.93 ± 1.03	1.96 ± 0.73	0.9368
	**PRSW (mmHg** **±** **SD)**			**Minimum dP/dt (mmHg/s** **±** **SD)**		
0	80.6 ± 17.2	77.2 ± 7.3	0.5784	−7,764 ± 955	−8,515 ± 1,128	0.1562
0.2475	90.4 ± 20.9	86.6 ± 15.1	0.6621	−8,309 ± 889	−9,130 ± 1,280	0.1490
0.825	103.8 ± 25.4	94.3 ± 13.6	0.3291	−8,415 ± 450	−8,782 ± 1,086	0.3943
2.475	117.3 ± 21.2	112.9 ± 21.1	0.6708	−8,383 ± 604	−8,549 ± 1,344	0.7528
8.25	119.5 ± 17.3	118.6 ± 17	0.9186	−8,264 ± 1,435	−8,304 ± 815	0.9405
	**Maximum dP/dt (mmHg/s** **±** **SD)**			**Tau (weiss-equation** **±** **SD)**		
0	7,514 ± 1,445	8,052 ± 1,528	0.4167	6.01 ± 0.73	6.29 ± 0.61	0.3889
0.2475	8,439 ± 1,686	9,120 ± 2,117	0.3944	5.74 ± 0.74	5.83 ± 0.42	0.7440
0.825	10,040 ± 1,609	9,787 ± 2,598	0.8858	5.44 ± 0.59	5.5 ± 0.4	0.7825
2.475	12,140 ± 1,597	13,027 ± 1,387	0.4199	5.3 ± 0.45	5.33 ± 0.54	0.8849
8.25	13,351 ± 1,130	14,074 ± 1,113	0.2565	5.43 ± 0.22	5.38 ± 0.69	0.8468

*PRSW, preload recruitable stroke work; EDPVR, end-diastolic pressure-volume relationship; n, number of mice; SD, standard deviation; p-values from unpaired Student's t-test between No PEEP and PEEP: Bold digits indicate significant p-values (< 0.05)*.

### The Influence of the Time Point of Calibration Using Hypertonic Saline Injection

Estimation of parallel-conductance by injecting hypertonic saline may be performed prior to or after PV recordings. We hypothesized that injection of hypertonic saline prior to PV-loop recordings may adversely affect murine cardiac function. Hence, we compared these two time points of hypertonic saline injections concerning their effects on PV analysis under β-adrenergic stimulation.

**Figure 2:**
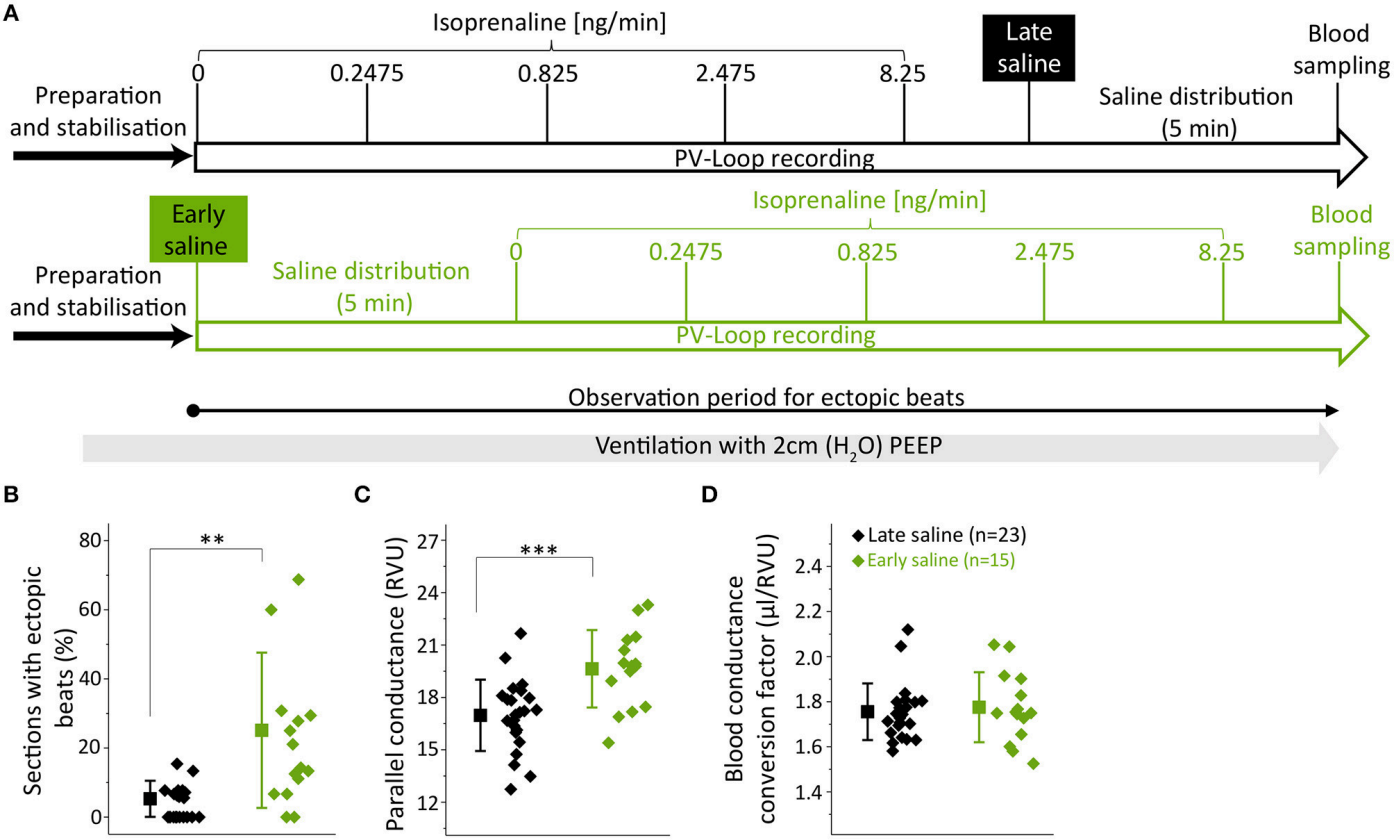
Early hypertonic saline calibration increased ectopic beat frequency and parallel-conductance. **(A)** Experimental design indicating the application time points of the hypertonic saline bolus either after (late saline, black) or prior to (early saline, green) PV loop recordings; **(B)** Analysis of ectopic beats showing the percentual proportion of sections with ectopic beats; **(C)** Parallel-conductance (Gp) calculated in groups of early or late saline application; **(D)** Blood conductance conversion factor for volume conversion (μl/RVU) at the end of experiments. Saline-bolus: One-time injection of 10 μl saline (15%) into the left femoral vein. Data presented as mean ± standard deviation from *n* = 23 (late saline) and *n* = 15 (early saline) mice. PEEP, positive end-expiratory pressure, ISO, Isoprenaline, RVU, Relative volume units; *n*, number of mice. **p < 0.01; ***p < 0.001: *p*-value of late saline vs. early saline in an unpaired Student's *t*-test.

Fifteen animals received 10 μl of 15% NaCl prior to (early saline group) and 23 animals after the PV recordings (late saline group) (Figure 2A). Except for the time point of saline application, protocols did not differ between groups (Figure 2A). Since PEEP ventilation proved beneficial, both groups were ventilated with 2 cmH_2_O PEEP (Figure 2A).

As we often observed ectopic beats during on-line visualization of PV recordings in the early saline group, ectopic beat frequency was quantified. The observation period for ectopic beats was defined over the time period depicted in Figure 2A and subsequently divided into sections of 1 min. If an ectopic beat occurred within a section, this section was defined as section with ectopic beat. The numbers of sections analyzed for ectopic beats was similar between the groups (15.5 ± 1.9 vs. 15.4 ± 1.5, *p* = 0.0744). Strikingly, early saline calibration induced a >5-fold increase in the occurrence of sections with ectopic beats as compared to late saline calibration (21.8 ± 20.0 vs. 4.2 ± 4.6%, *p* = 0.0043, Figure 2B) suggesting that the application of hypertonic saline operates as a pro-arrhythmic stimulus.

Concerning the actual calibration parameters, parallel-conductance was increased when early as compared to late saline injection was applied (19.6 ± 2.2 vs. 17.0 ± 2.0 RVU; *p* = 0.0008, Figure 2C). We checked for heart weight as a potential interfering variable, which was similar between groups (Table S2). Notably, the blood-conductance conversion factor at the end of the experiments did not differ between groups (late saline: 1.75 ± 0.30 μl/RVU, early saline: 1.77 ± 0.16 μl/RVU, *p* = 0.6813, Figure 2D), suggesting late saline does not alter blood-conductance if blood sampling is performed 5 min after saline injection.

**Figure 3:**
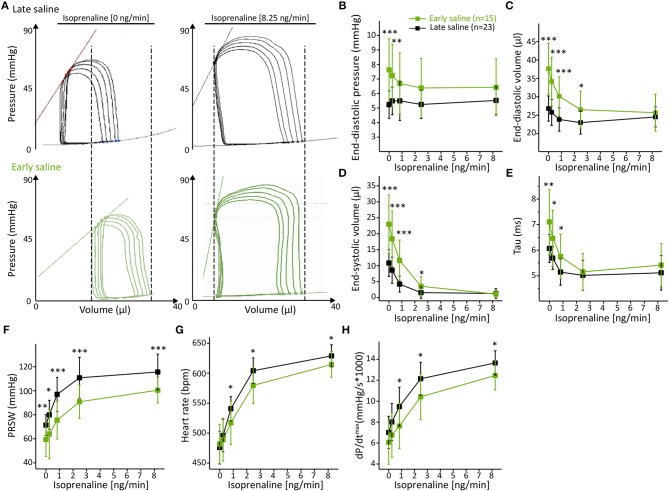
Early hypertonic saline calibration increased cardiac preload, reduced cardiac contractility and ameliorated β-adrenergic reserve. **(A)** Representative PV loops during inferior caval vein occlusion of the late saline group as compared to the early saline group; **(B)** Cardiac preload as depicted by end-diastolic pressure **(B)**, end-diastolic volume **(C)**, and end-systolic volume **(D)**; **(E)** Analysis of the diastolic relaxation constant Tau; **(F)** Analysis of systolic contractility (PRSW); **(G)** maximum dP/dt and **(H)** heart rate. Data presented as mean ± standard deviation from *n* = 23 (late saline group) and *n* = 15 (early saline group) mice. Abbreviations: ISO, Isoprenaline, PRSW, Preload Recruitable Stroke Work; *n*, number of mice. *p < 0.05; **p < 0.01; ***p < 0.001: *p*-value of Late saline vs. Early saline in an unpaired Student's *t*-test.

Analysis of basal left-ventricular function revealed multiple alterations between the two calibration regimens. Five minutes after early saline injection, PV loops showed a prominent right shift (Figure 3A) toward dilated chamber dimensions, similarly as in acute heart failure-like phenotypes with a 46% increase in end-diastolic pressure (P_ed_, *p* = 0.0007, Figure 3B), a 40% increase in end-diastolic volume (V_ed_, *p* < 0.0001, Figure 3C) and a 113% increase in end-systolic volume (V_es_, *p* = 0.0001, Figure 3D). The rise in volume load was accompanied by prolonged diastolic relaxation (Tau +17%, *p* = 0.0080, Figure 3E) and deteriorated cardiac contractility, as depicted by a 17% decrease in Preload Recruitable Stroke Work (PRSW, *p* = 0.0081, Figure 3F). Differences in cardiac chamber dimensions and diastolic relaxation diminished upon incremental β-adrenergic stimulation (Figures 3B–E). However, PRSW remained significantly impaired even at maximal β-adrenergic stimulation (Figure 3F), suggesting permanent effects of the hypertonic salt bolus independent of volume loading.

Although unaffected at basal conditions, the analysis of heart rate (Figure 3G) and maximum dP/dt (Figure 3H) revealed an alleviated adrenergic response as ISO-evoked increases in these parameters were also extenuated over the entire dose range. These results further emphasize that the adverse effects of early saline application on cardiac function are not transient.

To observe other potentially interfering parameters and conditions, we controlled for e.g., body temperature during PV recordings, as well as for hypertrophy indices, which did not differ between groups (Table S2). Detailed values for each analyzed parameter are given in Table S3.

### The Impact of End-Systolic Pressure-Spikes

**Figure 4:**
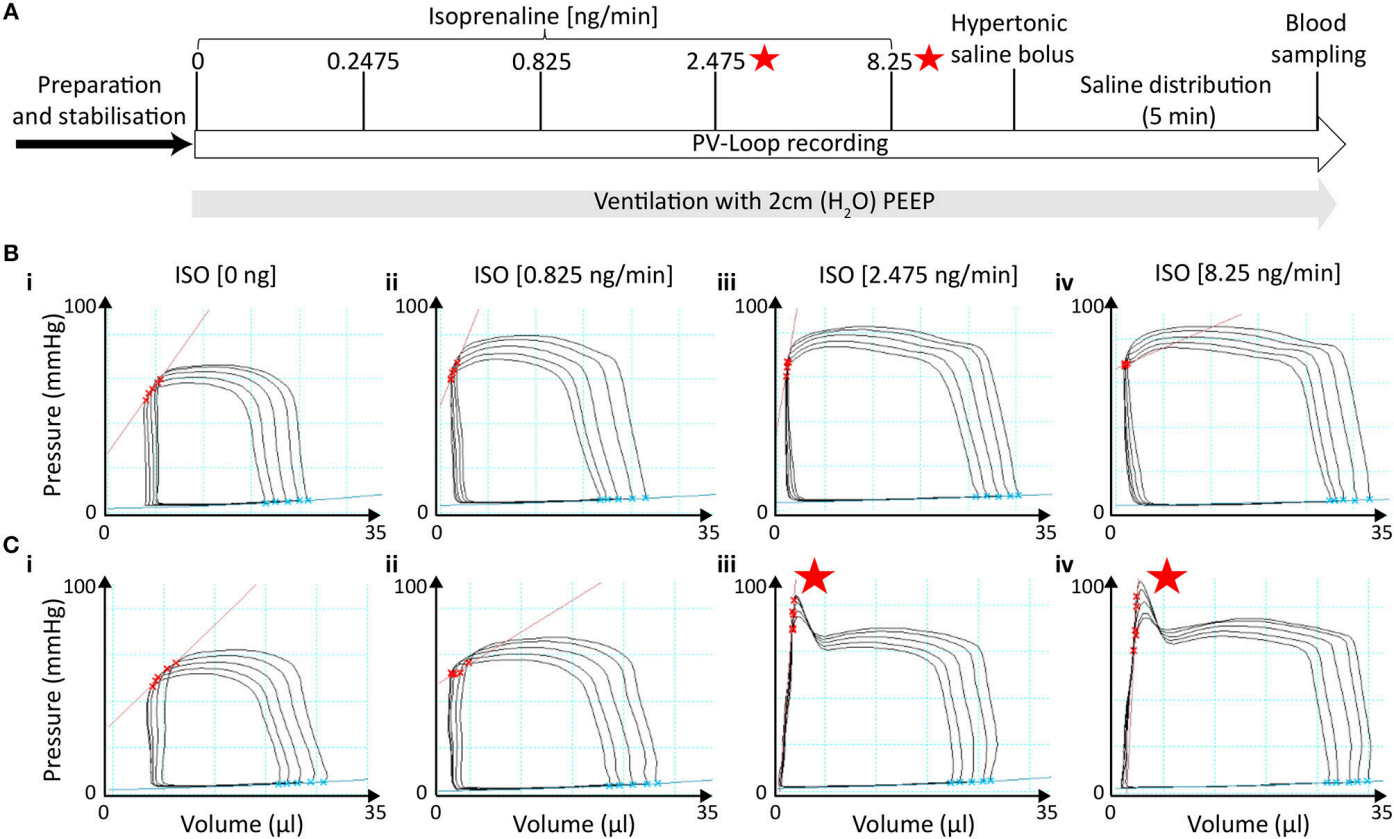
End-systolic pressure-spikes under β-adrenergic stimulation occurred in measurements without initial signs of catheter entrapment. **(A)** Time of ESPS occurrence during the measurements labeled by red stars. (**B**_i−iv_) Representative PV loops during ICV occlusion under incremental ISO stimulation in a measurement in which no ESPS were detected at any concentration. (**C**_i−iv_) Representative PV loops during ICV occlusion in a measurement in which ESPS were detected during stimulation with 2.475 ng/min ISO or higher. ESPS, End-systolic pressure-spikes; ISO, Isoprenaline.

End-systolic pressure-spikes (ESPS) describe artificial pressure increases at the very end of the systolic ejection phase. In the protocol with 2 cmH_2_O PEEP-ventilation and late saline calibration, ESPS were observed in 6 out of 23 recordings but only at stimulation with doses at or above 2.475 ng/min Isoprenaline (Figure 4A). Hence, we divided PV loop recordings obtained with this protocol into two groups, which either presented ESPS upon β-adrenergic stimulation (*n* = 6) or not (*n* = 17) and examined the effect of ESPS on PV parameters.

**Figure 5:**
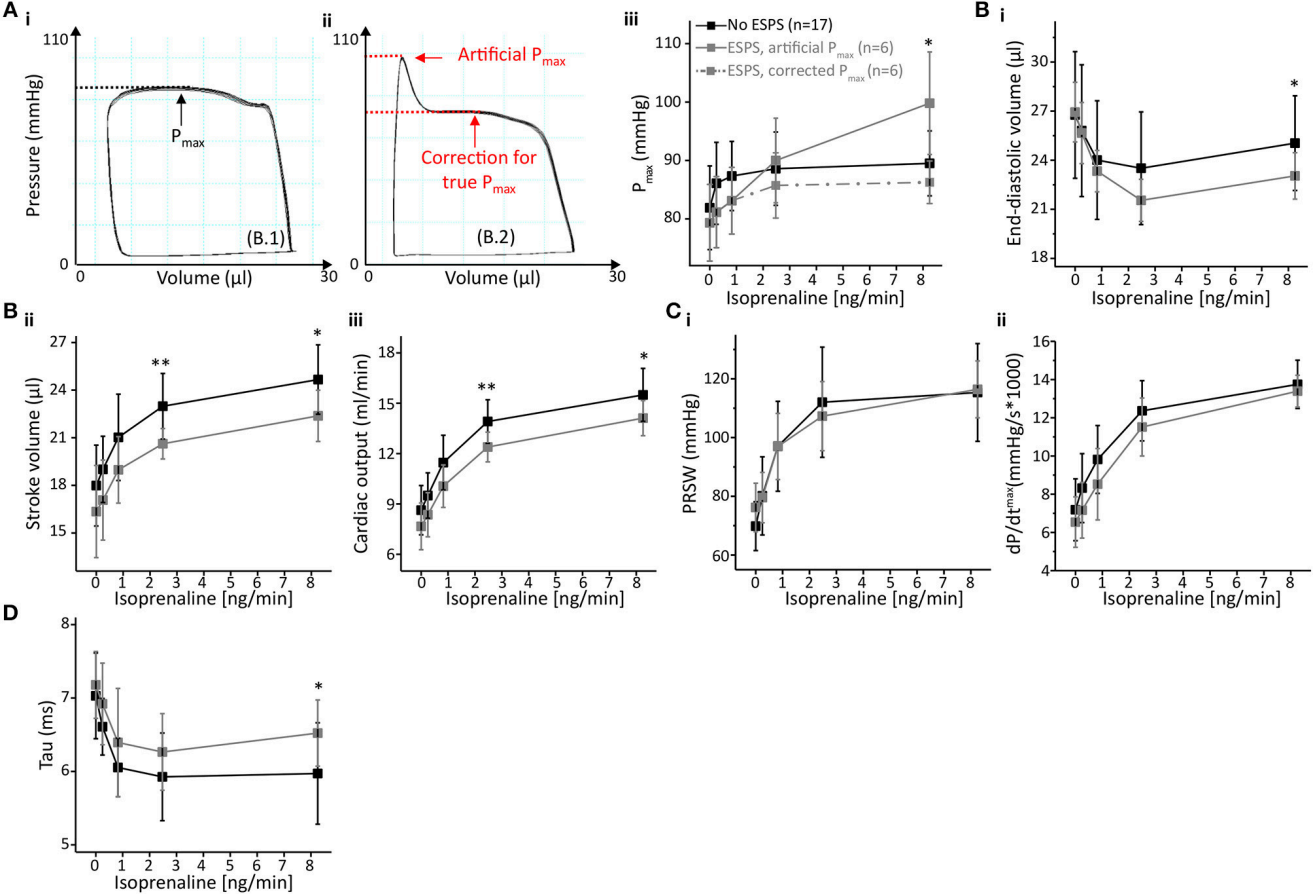
End-systolic pressure-spikes impaired volume detection and diastolic relaxation but did not affect cardiac contractility. **(A)** Impact of ESPS on pressure detection with representative PV loops either without (**A**_i_) or with ESPS (**A**_ii_). Maximum pressure (P_max_) could be corrected as depicted by red arrows and lines (**A**_ii_); a comparison between P_max_ in both groups, including corrected P_max_ for measurements with ESPS (dashed line) is shown in (**A**_iii_). **(B)** Volumetric parameters end-diastolic volume (**B**_i_), stroke volume (**B**_ii_) and cardiac output (**B**_iii_). **(C)** Analysis of cardiac contractility showing preload recruitable stroke work (PRSW) (**C**_i_) and maximum dP/dt (**C**_ii_). **(D)** Diastolic relaxation constant Tau. Data presented as mean ± standard deviation from *n* = 17 (No ESPS group) and *n* = 6 (ESPS group) mice. ESPS, End-systolic pressure-spike; ISO, Isoprenaline; *n*, number of mice. *p < 0.05; **p < 0.01: *p*-values No ESPS vs. ESPS in an unpaired Student's *t*-test.

Notably, PV recordings in mice that developed ESPS at higher ISO doses (≥2.475 ng/min) were otherwise morphologically not distinguishable at basal levels or at the lowest ISO dose (0.825 ng/min) from those that did not develop ESPS (Figures 4B,C). Furthermore, both groups showed comparable cardiac function at basal levels (Figure 5; Table S4), indicating similar catheter position prior to catecholamine stimulation.

ESPS artificially increase pressure near end-systole. Hence, PV loop analysis of recordings depicting ESPS comprises an artificially increased maximum pressure (P_max_, Figure 5A). However, this “artificial P_max_” could be corrected to “true P_max_” by adjusting P_max_ to the maximum value during auxotonic contractions, as depicted in Figure 5A. Following this maneuver, alterations in P_max_ could be extinguished (Figure 5A).

Other pressure-derived contractility parameters that are potentially affected by increased pressure values close to the end-systole include diastolic relaxation parameters. Hence, we checked for alterations in diastolic function. Interestingly, ESPS had opposite effects on the two parameters characterizing diastolic relaxation: analysis of Tau in PV recordings depicting ESPS at 8.25 ng/min ISO indicated a deceleration of active diastolic relaxation by 11% (*p* = 0.0445, Figure 5D), whereas minimum dP/dt values revealed an acceleration by 18.4% (dP/dt_min_, *p* = 0.0453, Table S5). ESPS were associated with slower relaxation in dP/dt_min_ at 0.2475 and 0.825 ng/min ISO (Table S5).

In addition, ESPS also impaired volume-related parameters (Figure 5B), as depicted by an about 8.5% decrease in end-diastolic volume (*p* = 0.0611 and *p* = 0.0407 at 2.475 and 8.25 ng/min ISO, respectively). Stroke volume was reduced by 9–10% (*p* = 0.0011 and *p* = 0.0112 at 2.475 and 8.25 ng/min ISO, respectively) as well as cardiac output (*p* = 0.0066 and *p* = 0.0177 at 2.475 and 8.25 ng/min ISO, respectively). With regards to systolic cardiac function, we could not observe differences in parameters of cardiac contractility (Figure 5C), neither in preload-independent PRSW, nor in preload dependent dP/dt_max_.

We also checked for body-weight, hypertrophy indices and body temperature during measurements, which were comparable between groups (Table S4). This further suggests these variables do not determine the likelihood of ESPS-development. Exact values for each PV parameter are given in Table S5.

## Discussion

The salient findings of our study about key methodological factors in murine pressure-volume (PV) analysis under β-adrenergic stimulation were that (i) ventilation with 2 cmH_2_O positive end-expiratory pressure prevents pulmonary lesions and does not alter hemodynamic conditions, (ii) injections of hypertonic saline prior to pressure-volume recordings deteriorate cardiac function and facilitate the occurrence of ectopic beats, and (iii) end-systolic pressure-spikes (ESPS) impair not only pressure-derived, but also volumetric parameters and diastolic relaxation.

### Positive End-Expiratory Pressure

PEEP-ventilation most prominently protected mice from exacerbated increases of peak inspiratory pressure and macroscopic pulmonary lesions. Both could be observed most likely due to PEEP-ventilation reducing pulmonary atelectasis, subsequently improving pulmonary compliance and gas exchange as known in man and mice (19, 20). However, ventilation with 15 cmH_2_O PEEP in pigs can affect cardiac output due to preload reduction (21). This phenomenon is present during closed-chest conditions, but to a lesser degree also in open-chest instrumentation (21). However, ventilation with a moderate PEEP of 2 cmH_2_O during open-chest PV analysis did not affect murine left-ventricular function in our protocol. We therefore regard ventilation using 2 cmH_2_O PEEP as being safe in the context of murine open-chest PV analysis. Though ventilation with a PEEP of 5 cmH_2_O has already been used during open-chest PV analysis in mice, PEEP-ventilation has not been evaluated concerning its impacts on murine cardiac function in previous studies (10). However, it remains to be investigated whether and to which extent PEEP ventilation may affect murine cardiac performance in closed-chest PV analysis and/or in murine models of heart failure, e.g., myocardial infarction, pressure overload or myocarditis (22).

### Hypertonic Saline Calibration

Injecting hypertonic saline to calibrate for parallel-conductance 5 min prior to PV recordings deteriorated basal cardiac function as compared to its injection after PV recordings. Animals of the early saline group presented in an acute dilative heart failure-like phenotype depicting right shifted PV recordings and elevated end-diastolic pressure (P_ed_). End-diastolic volume (V_ed_) and end-systolic volume (V_es_) alone could have been artificially enlarged by the 15% NaCl bolus increasing blood conductance. However, higher P_ed_ points toward true volume overloading, as P_ed_ is known to rise proportionally with cardiac preload (23). Potentially, early hyperosmolar saline application increased intra-vascular volume and concomitantly impaired also PRSW, a measure of preload-independent cardiac contractility, resulting in severe accumulation of blood proximal to the ventricle. These results are in line with findings in which impairment of maximum dP/dt was attributable to hypertonic saline injections in mice (6). As incremental β-adrenergic stimulation increased cardiac contractility in both groups, differences in cardiac volumes leveled off.

However, early hypertonic saline calibration also had non-recoverable effects on murine cardiac function, which were observed as permanent impairment of preload-independent contractility as well as in alleviated chronotropic and inotropic ISO-response. Possible explanations for the adverse effects on cardiac function include persisting effects following acute volume overload as well as severe electrolyte imbalance, such as hypernatremia and/or hyperchloremia. An increase in extracellular Na^+^ levels may aggravate the electrochemical driving force for the Na^+^/Ca^2x^ Exchanger (NCX), thereby triggering trans-sarcolemmal Ca^2+^-efflux, subsequently depleting sarcoplasmic reticulum stores, which can result in depressed contractility (24, 25). Other mechanisms could include the influence of high extracellular Na^+^ on sodium-conducting channels that fine-tune cardiac contractility, such as TRPM4, which is known to be active under conditions of β-adrenergic stimulation (15, 26, 27). As animals had reached a new post-calibration steady state 5 min after early hypertonic saline calibration, we assume the observed effects of early hypertonic saline to be persistent. However, we cannot fully exclude that animals may further recover after a prolonged time of saline distribution prior to isoprenaline-challenge.

Moreover, dilated chamber dimensions that were observed in conditions applying early saline calibration increased ectopic beat frequency >5-fold most likely via elevation of intra-ventricular wall stretch, which is known to induce ectopic beats through stretch-activated channels (28–31). Furthermore, findings from contractility analysis in canine and human hearts, in which the relaxation constant Tau positively correlates with end-systolic volume (V_es_) (32, 33), indicate that Tau was increased by enlarged intra-ventricular volumes. Minimum dP/dt (dP/dt_min_) was not altered in our study, as it is known to correlate with end-systolic pressure (34, 35), which was indifferent between groups (data not shown).

Interestingly, G_p_ was higher in the early saline group, though heart weight, which is known to positively correlate with G_p_ (36), was unaltered between groups. However, alterations in contractility during the hypertonic saline's left ventricular wash-in phase can increase G_p_ (36). These alterations in contractility were potentially less pronounced in the late saline group, as 8.25 ng/min ISO had already induced a higher contractile state at the time point of saline calibration.

### End-Systolic Pressure-Spikes

End-systolic pressure-spikes (ESPS) appeared following β-adrenergic stimulation using 2.475 ng/min ISO or higher. ESPS most likely resulted from a direct contact between the pressure transducer and papillary muscles and/or the left ventricular wall, as known from PV analysis and simultaneous echocardiography in rats (37). Strikingly, ESPS would potentially be rectifiable by catheter repositioning. However, in a longitudinal protocol, one cannot reposition the catheter if ESPS occur, since repositioning would significantly alter the parallel conductance within the catheter's electrical field. Thus, additional hypertonic saline calibrations would be required in recordings with ESPS, but not in those without ESPS, which would markedly affect the measurements. As one cannot reposition the catheter in this setting, we analyzed the impact of ESPS on PV parameters in order to optimize data analysis and reproducibility in this context.

In addition to the anticipated effects on pressure-derived parameters, which in case of maximum pressure were easily rectifiable, the occurrence of ESPS also impaired volume detection. As known from rats, the occurrence of ESPS correlates with a rather lateral catheter position (37), which increases the proportion of less conductive myocardium in the catheters electrical field. This may explain the reduced volume detection.

Unlike others in rats (37), we could not observe effects of ESPS on maximum dP/dt in PV analysis in mice. This is in line with the common understanding that dP/dt_max_ characterizes isovolumetric contraction (23) and ESPS in our protocol occurred at the end of auxotonic contraction. However, ESPS impaired the calculation of the diastolic relaxation parameters Tau and dP/dt_min_. The calculation of both parameters positively correlates with the value for end-systolic pressure (34, 35, 38), which was artificially increased by the pressure spike near end-systole in PV recordings depicting ESPS. This may explain why ESPS artificially induced the contradictory finding of higher values, thus faster relaxation, in dP/dt_min_, and higher values, thus slower relaxation, in Tau.

### Limitations

In our evaluation of PEEP-ventilation, we did not quantify pulmonary damage on a histological level by e.g., quantifying the formation of atelectasis. However, macroscopic hemorrhages, which were completely absent in PEEP-ventilated animals, depict even more severe forms of pulmonary injury. In line with this, others have previously shown protective effects of PEEP-ventilation on a histological level in mice (39), but not in the context of PV analysis. Moreover, we used the femoral vein as the injection site for hypertonic saline in our protocol, though estimation of parallel-conductance (G_p_) by injecting hypertonic saline has previously been reported for the jugular vein or pulmonary artery (6, 7). Nonetheless, our G_p_-values using the femoral vein as the central venous injection site are in the range of those reported using jugular vein injections (6, 7, 9).

### Conclusions

(i) Our results support the use of PEEP ventilation in murine open-chest pressure-volume (PV) analysis, as it prevents pulmonary lesions. (ii) They further endorse not to calibrate for parallel-conductance by injecting hypertonic saline prior to the actual PV recordings, due to its multiple adverse effects on murine cardiac function. (iii) We point out to consider that end-systolic pressure-spikes (ESPS), which may arise during incremental β-adrenergic stimulation, adversely affect specific parameters in murine PV analysis.

## Author Contributions

LB, MF, and JCL conceived and planned the experiments. LB carried out the experiments. JCL and MF supervised the study. SS, RM, and DL contributed to the interpretation of the results. LB, MF, and JCL wrote the manuscript. All authors provided critical feedback and helped shape the research, analysis and manuscript.

### Conflict of Interest Statement

The authors declare that the research was conducted in the absence of any commercial or financial relationships that could be construed as a potential conflict of interest.

## References

[1] Georgakopoulos D Mitzner WA Chen CH Byrne BJ Millar HD Hare JM. *In vivo* murine left ventricular pressure-volume relations by miniaturized conductance micromanometry. Am J Physiol. (1998) 274(4 Pt 2):H1416–22. 10.1152/ajpheart.1998.274.4.H1416 9575947

[2] Cingolani OH Kass DA. Pressure-volume relation analysis of mouse ventricular function. Am J Physiol Heart Circ Physiol. (2011) 301:H2198–206. 10.1152/ajpheart.00781.2011 21926344

[3] Lindsey ML Kassiri Z Virag JAI de Castro Bras LE Scherrer-Crosbie M. Guidelines for measuring cardiac physiology in mice. Am J Physiol Heart Circ Physiol. (2018) 314:H733–52. 10.1152/ajpheart.00339.2017 29351456 PMC5966769

[4] Baan J van der Velde ET de Bruin HG Smeenk GJ Koops J van Dijk AD. Continuous measurement of left ventricular volume in animals and humans by conductance catheter. Circulation. (1984) 70:812–23. 10.1161/01.CIR.70.5.812 6386218

[5] Lankford EB Kass DA Maughan WL Shoukas AA. Does volume catheter parallel conductance vary during a cardiac cycle? Am J Physiol. (1990) 258(6 Pt 2):H1933–42. 10.1152/ajpheart.1990.258.6.H1933 2360681

[6] Georgakopoulos D Kass DA. Estimation of parallel conductance by dual-frequency conductance catheter in mice. Am J Physiol Heart Circ Physiol. (2000) 279:H443–50. 10.1152/ajpheart.2000.279.1.H443 10899085

[7] Nielsen JM Kristiansen SB Ringgaard S Nielsen TT Flyvbjerg A Redington AN. Left ventricular volume measurement in mice by conductance catheter: evaluation and optimization of calibration. Am J Physiol Heart Circ Physiol. (2007) 293:H534–40. 10.1152/ajpheart.01268.2006 17384122

[8] Yang B Larson DF Beischel J Kelly R Shi J Watson RR. Validation of conductance catheter system for quantification of murine pressure-volume loops. J Invest Surg. (2001) 14:341–55. 10.1080/089419301753435710 11905502

[9] Pacher P Nagayama T Mukhopadhyay P Batkai S Kass DA. Measurement of cardiac function using pressure-volume conductance catheter technique in mice and rats. Nat Protoc. (2008) 3:1422–34. 10.1038/nprot.2008.138 18772869 PMC2597499

[10] Townsend D Measuring pressure volume loops in the mouse. J Vis Exp. (2016) e53810 10.3791/53810 PMC4942027 27166576

[11] Lips DJ van der Nagel T Steendijk P Palmen M Janssen BJ van Dantzig JM. Left ventricular pressure-volume measurements in mice: comparison of closed-chest versus open-chest approach. Basic Res Cardiol. (2004) 99:351–9. 10.1007/s00395-004-0476-5 15309414

[12] Oosterlinck W Vanderper A Flameng W Herijgers P. Glucose tolerance and left ventricular pressure-volume relationships in frequently used mouse strains. J Biomed Biotechnol. (2011) 2011:281312. 10.1155/2011/281312 21318112 PMC3035009

[13] Westermann D Knollmann BC Steendijk P Rutschow S Riad A Pauschinger M. Diltiazem treatment prevents diastolic heart failure in mice with familial hypertrophic cardiomyopathy. Eur J Heart Fail. (2006) 8:115–21. 10.1016/j.ejheart.2005.07.012 16214409

[14] Nemoto S DeFreitas G Mann DL Carabello BA. Effects of changes in left ventricular contractility on indexes of contractility in mice. Am J Physiol Heart Circ Physiol. (2002) 283:H2504–10. 10.1152/ajpheart.0765.2001 12427596

[15] Mathar I Kecskes M Van der Mieren G Jacobs G Camacho Londono JE Uhl S Increased beta-adrenergic inotropy in ventricular myocardium from Trpm4^−/−^ mice. Circ Res. (2014) 114:283–94. 10.1161/CIRCRESAHA.114.302835 24226423

[16] Gargiulo S Greco A Gramanzini M Esposito S Affuso A Brunetti A. Mice anesthesia, analgesia, and care, part I: anesthetic considerations in preclinical research. ILAR J. (2012) 53:E55–69. 10.1093/ilar.53.1.55 23382271

[17] Glower DD Spratt JA Snow ND Kabas JS Davis JW Olsen CO. Linearity of the frank-starling relationship in the intact heart: the concept of preload recruitable stroke work. Circulation. (1985) 71:994–1009. 10.1161/01.CIR.71.5.994 3986986

[18] Weiss JL Frederiksen JW Weisfeldt ML. Hemodynamic determinants of the time-course of fall in canine left ventricular pressure. J Clin Invest. (1976) 58:751–60. 10.1172/JCI108522 956400 PMC333234

[19] Rusca M Proietti S Schnyder P Frascarolo P Hedenstierna G Spahn DR. Prevention of atelectasis formation during induction of general anesthesia. Anesth Analg. (2003) 97:1835–9. 10.1213/01.ANE.0000087042.02266.F6 14633570

[20] Thammanomai A Hamakawa H Bartolak-Suki E Suki B. Combined effects of ventilation mode and positive end-expiratory pressure on mechanics, gas exchange and the epithelium in mice with acute lung injury. PLoS ONE. (2013) 8:e53934. 10.1371/journal.pone.0053934 23326543 PMC3541132

[21] Kubitz JC Annecke T Kemming GI Forkl S Kronas N Goetz AE. The influence of positive end-expiratory pressure on stroke volume variation and central blood volume during open and closed chest conditions. Eur J Cardiothorac Surg. (2006) 30:90–5. 10.1016/j.ejcts.2006.04.008 16723238

[22] Bacmeister L Schwarzl M Warnke S Stoffers B Blankenberg S Westermann D. Inflammation and fibrosis in murine models of heart failure. Basic. Res. Cardiol. (2019) 114:19. 10.1007/s00395-019-0722-5 30887214

[23] Burkhoff D Mirsky I Suga H. Assessment of systolic and diastolic ventricular properties via pressure-volume analysis: a guide for clinical, translational, and basic researchers. Am J Physiol Heart Circ Physiol. (2005) 289:H501–12. 10.1152/ajpheart.00138.2005 16014610

[24] Aronsen JM Swift F Sejersted OM. Cardiac sodium transport and excitation-contraction coupling. J Mol Cell Cardiol. (2013) 61:11–9. 10.1016/j.yjmcc.2013.06.003 23774049

[25] Ottolia M Torres N Bridge JH Philipson KD Goldhaber JI. Na/Ca exchange and contraction of the heart. J Mol Cell Cardiol. (2013) 61:28–33. 10.1016/j.yjmcc.2013.06.001 23770352 PMC3730535

[26] Uhl S Mathar I Vennekens R Freichel M. Adenylyl cyclase-mediated effects contribute to increased isoprenaline-induced cardiac contractility in TRPM4-deficient mice. J Mol Cell Cardiol. (2014) 74:307–17. 10.1016/j.yjmcc.2014.06.007 24972051

[27] Freichel M Berlin M Schürger A Mathar I Bacmeister L Medert R TRP channels in the heart. In: Emir TLR, editor. Neurobiology of TRP Channels. 1. New York, NY: CRC Press (2017). p. 149–86. 29356479

[28] Franz MR Burkhoff D Yue DT Sagawa K. Mechanically induced action potential changes and arrhythmia in isolated and *in situ* canine hearts. Cardiovasc Res. (1989) 23:213–23. 10.1093/cvr/23.3.213 2590905

[29] Franz MR Cima R Wang D Profitt D Kurz R. Electrophysiological effects of myocardial stretch and mechanical determinants of stretch-activated arrhythmias. Circulation. (1992) 86:968–78. 10.1161/01.CIR.86.3.968 1381296

[30] Kamkin A Kiseleva I Isenberg G. Stretch-activated currents in ventricular myocytes: amplitude and arrhythmogenic effects increase with hypertrophy. Cardiovasc Res. (2000) 48:409–20. 10.1016/S0008-6363(00)00208-X 11090836

[31] Taggart P Sutton P Lab M. Interaction between ventricular loading and repolarisation: relevance to arrhythmogenesis. Br Heart J. (1992) 67:213–5. 10.1136/hrt.67.3.213 1554538 PMC1024793

[32] Courtois M Mechem CJ Barzilai B Ludbrook PA. Factors related to end-systolic volume are important determinants of peak early diastolic transmitral flow velocity. Circulation. (1992) 85:1132–8. 10.1161/01.CIR.85.3.1132 1537111

[33] Udelson JE Bacharach SL Cannon RO III Bonow RO. Minimum left ventricular pressure during beta-adrenergic stimulation in human subjects. Evidence for elastic recoil and diastolic “suction” in the normal heart. Circulation. (1990) 82:1174–82. 10.1161/01.CIR.82.4.1174 1976048

[34] Cohn PF Liedtke AJ Serur J Sonnenblick EH Urschel CW. Maximal rate of pressure fall (peak negative dP-dt) during ventricular relaxation. Cardiovasc Res. (1972) 6:263–7. 10.1093/cvr/6.3.263 5035596

[35] Weisfeldt ML Scully HE Frederiksen J Rubenstein JJ Pohost GM Beierholm E. Hemodynamic determinants of maximum negative dP-dt and periods of diastole. Am J Physiol. (1974) 227:613–21. 10.1152/ajplegacy.1974.227.3.613 4413651

[36] Feldman MD Erikson JM Mao Y Korcarz CE Lang RM Freeman GL. Validation of a mouse conductance system to determine LV volume: comparison to echocardiography and crystals. Am J Physiol Heart Circ Physiol. (2000) 279:H1698–707. 10.1152/ajpheart.2000.279.4.H1698 11009457

[37] Wei AE Maslov MY Pezone MJ Edelman ER Lovich MA. Use of pressure-volume conductance catheters in real-time cardiovascular experimentation. Heart Lung Circ. (2014) 23:1059–69. 10.1016/j.hlc.2014.04.130 24954709 PMC4241179

[38] Leite-Moreira AF Gillebert TC. Nonuniform course of left ventricular pressure fall and its regulation by load and contractile state. Circulation. (1994) 90:2481–91. 10.1161/01.CIR.90.5.2481 7955206

[39] Cagle LA Franzi LM Linderholm AL Last JA Adams JY Harper RW. Effects of positive end-expiratory pressure and recruitment maneuvers in a ventilator-induced injury mouse model. PLoS ONE. (2017) 12:e0187419. 10.1371/journal.pone.0187419 29112971 PMC5675408

