# Supplementary material for: Assessment of PEEP-Ventilation and the Time Point of Parallel-Conductance Determination for Pressure-Volume Analysis Under β-Adrenergic Stimulation in Mice
Source: Front Cardiovasc Med. 2019 Apr 10;6:36. doi: 10.3389/fcvm.2019.00036 (PMC6499229; doi:10.3389/fcvm.2019.00036)
Supplement: Supplementary file 1 [file Data_Sheet_1.pdf]

Supplemental information

Assessment of PEEP-ventilation and the time point of parallel-conductance determination for pressure-volume analysis under β-adrenergic stimulation in mice.

Bacmeister L[1,2*], Segin S[1,2], Medert R[1,2], Lindner, D[3,4], Freichel M[1,2] and Camacho Londoño JE[1,2]

1 Pharmakologisches Institut, Ruprecht-Karls-Universität Heidelberg, 69120 Heidelberg, Germany

3 DZHK (German Centre for Cardiovascular Research), partner site Heidelberg/Mannheim, Germany

3,*(current address) Universitäres Herzzentrum Hamburg, Experimentelle und Molekulare Kardiologie, 20251 Hamburg, Germany

4 DZHK (German Centre for Cardiovascular Research), partner site Hamburg/Kiel/Lübeck, Germany

Correspondence to: Lucas Bacmeister (lucas.bacmeister@pharma.uni-heidelberg.de and Juan E. Camacho Londoño (juan.londono@pharma.uni-heidelberg.de), INF-366, D-69120 Heidelberg, Germany, Tel.: +49-6221-54-86863, Fax: +49-6221-54-8644

Supplemental Figures

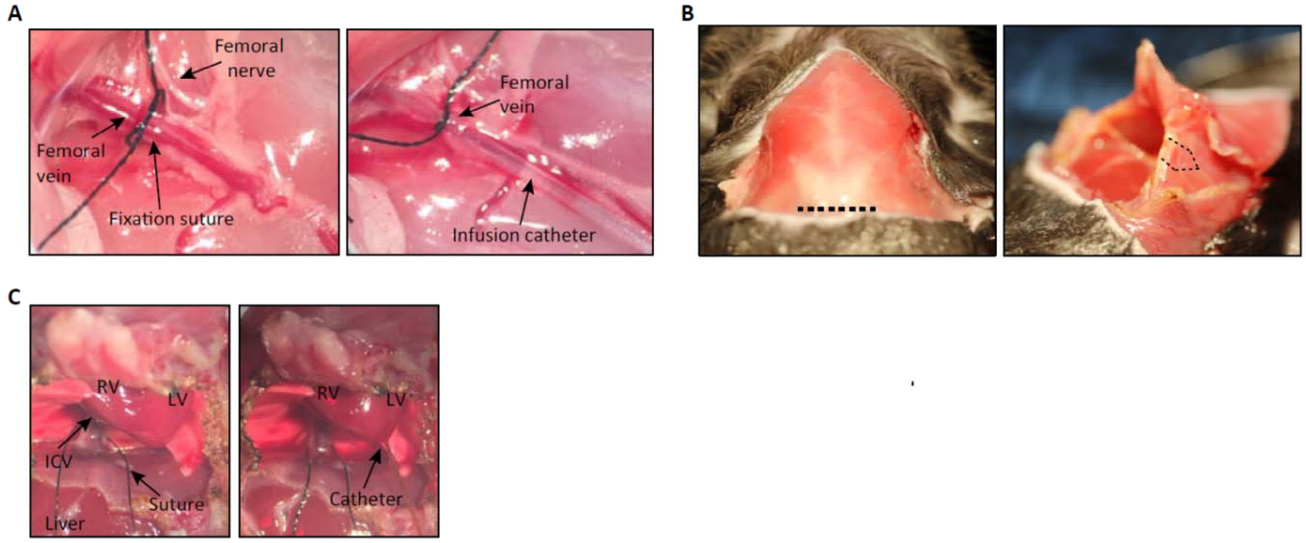

Figure S1. Surgical preparation for open-chest pressure-volume recordings. (**A**) Intra-operative picture of the femoral vein depicting the blunted vessel before (left panel) and after (right panel) cannulation with a polyethylene tube. (**B**) First steps of the thoracotomy showing the incision lines through abdominal muscles (left panel) and the ribs (right panel). (**C**) Intra-operative view on the dorsal thorax after thoracotomy depicting the occlusion suture beneath the inferior cava vein (ICV) before (left panel) and after (right panel) catheter insertion. Abbreviations: RV, Right ventricle and LV, Left ventricle.

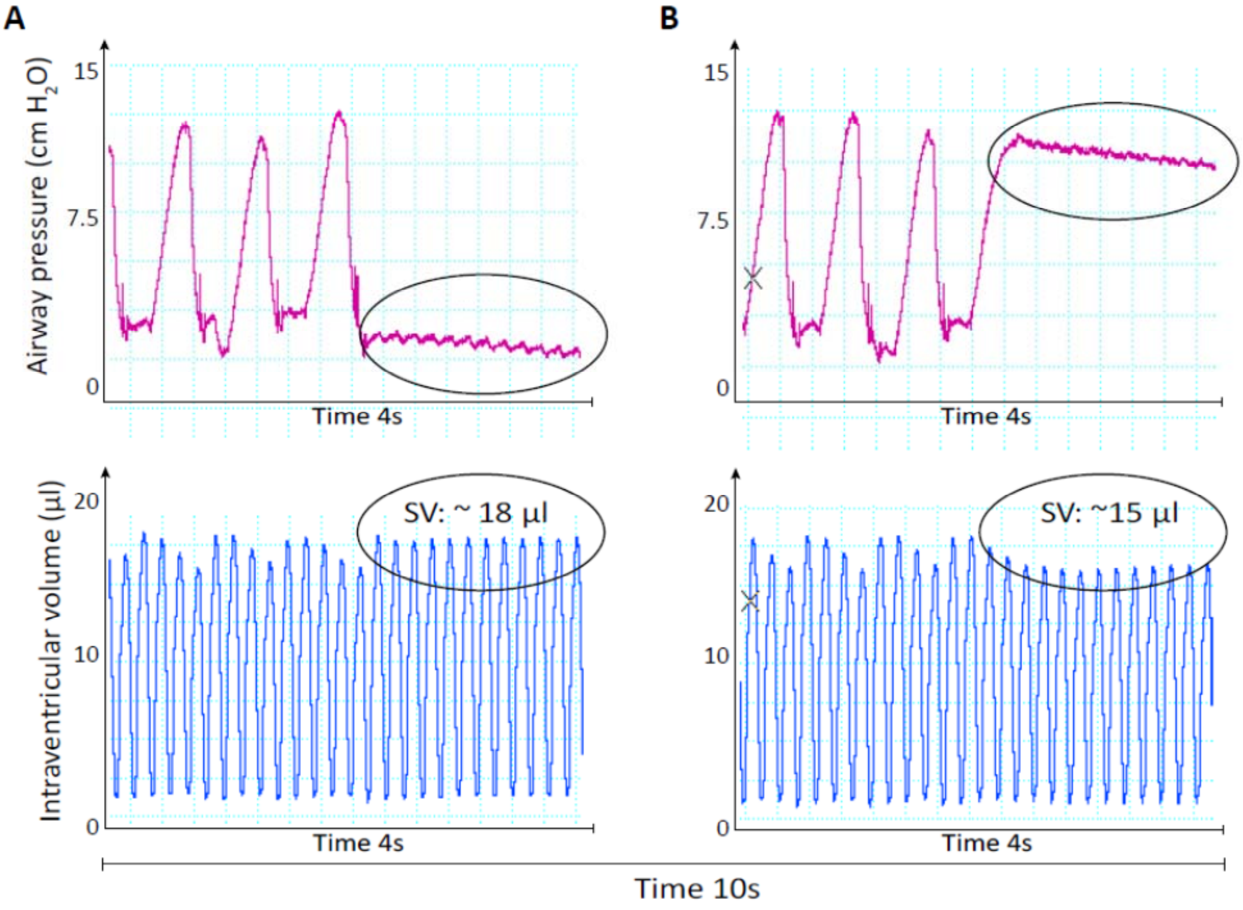

Figure S2. Significance of end-expirational ventilation stops. Representative (**A**) end-expirational and (**B**) end-inspirational ventilation stops from the same animal within 10s showing a typical decrease in stroke volume (SV) during an end-inspirational ventilation-stop. Upper traces show the airway pressure and the lower traces depict the intra-ventricular volume.

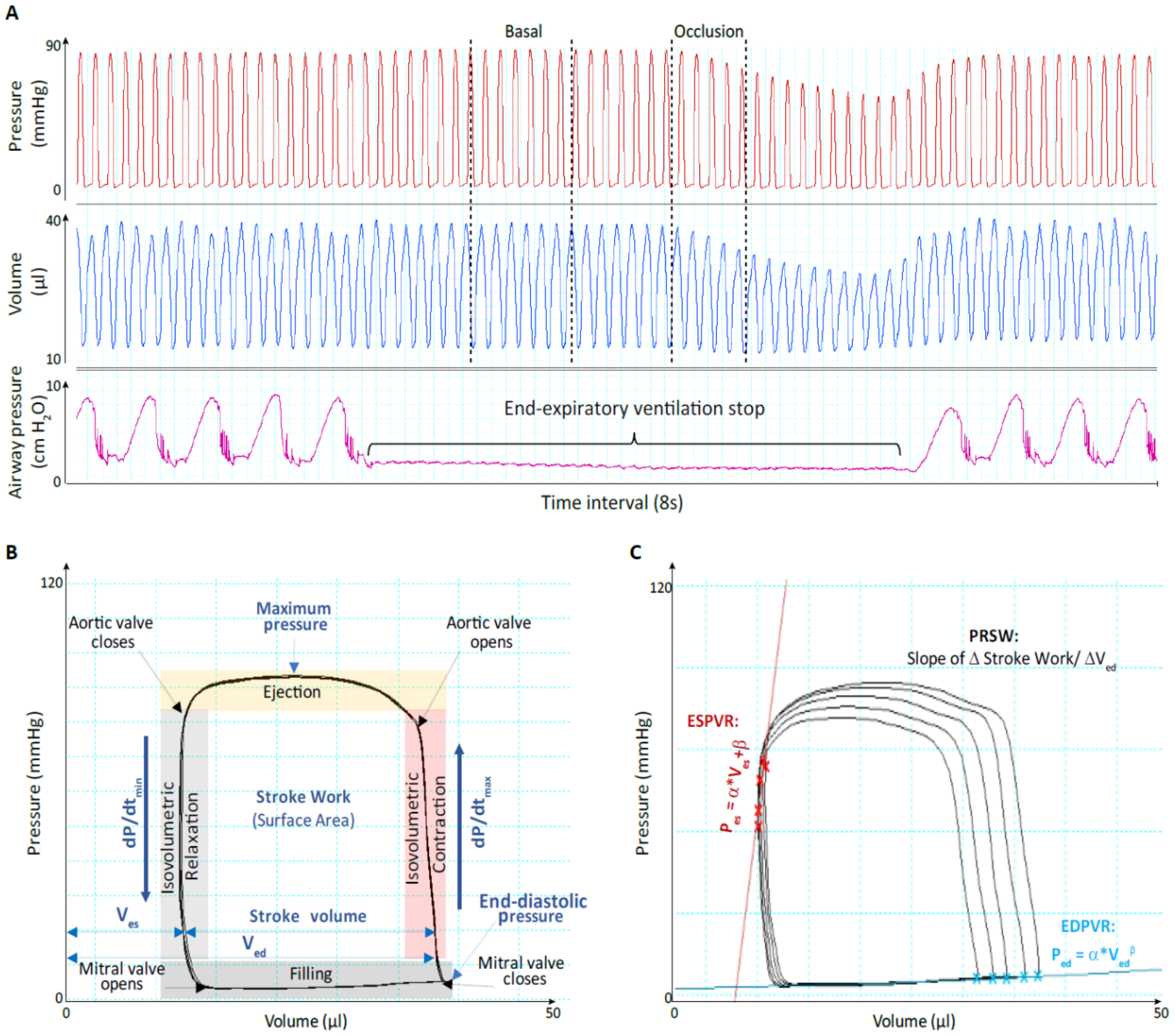

Figure S3. Representative pressure-volume analysis. (**A**) Representative traces of intra-ventricular pressure (upper traces), volume (middle traces) and airway pressure (lower traces). Dashed lines in (**A**) delimit the analysis of basal PV recordings and those during preload reduction evoked by caval vein occlusion. For the analysis of preload independent parameters, strictly the first five consecutive loops with decreased preload were selected. (**B** and **C**) Pressure-volume recordings and corresponding parameters during basal measurement (**B**) or preload-reduction (**C**) derived from areas labelled in (**A**). Abbreviations: dP/dt_{min}: Minimum dP/dt, dP/dt_{max}: Maximum dP/dt, V_{es}: End-systolic Volume, V_{ed}: End-diastolic Volume, ESPVR: End-systolic pressure-volume relationship, PRSW: Preload Recruitable Stroke Work, EDPVR: End-diastolic pressure-volume relationship.

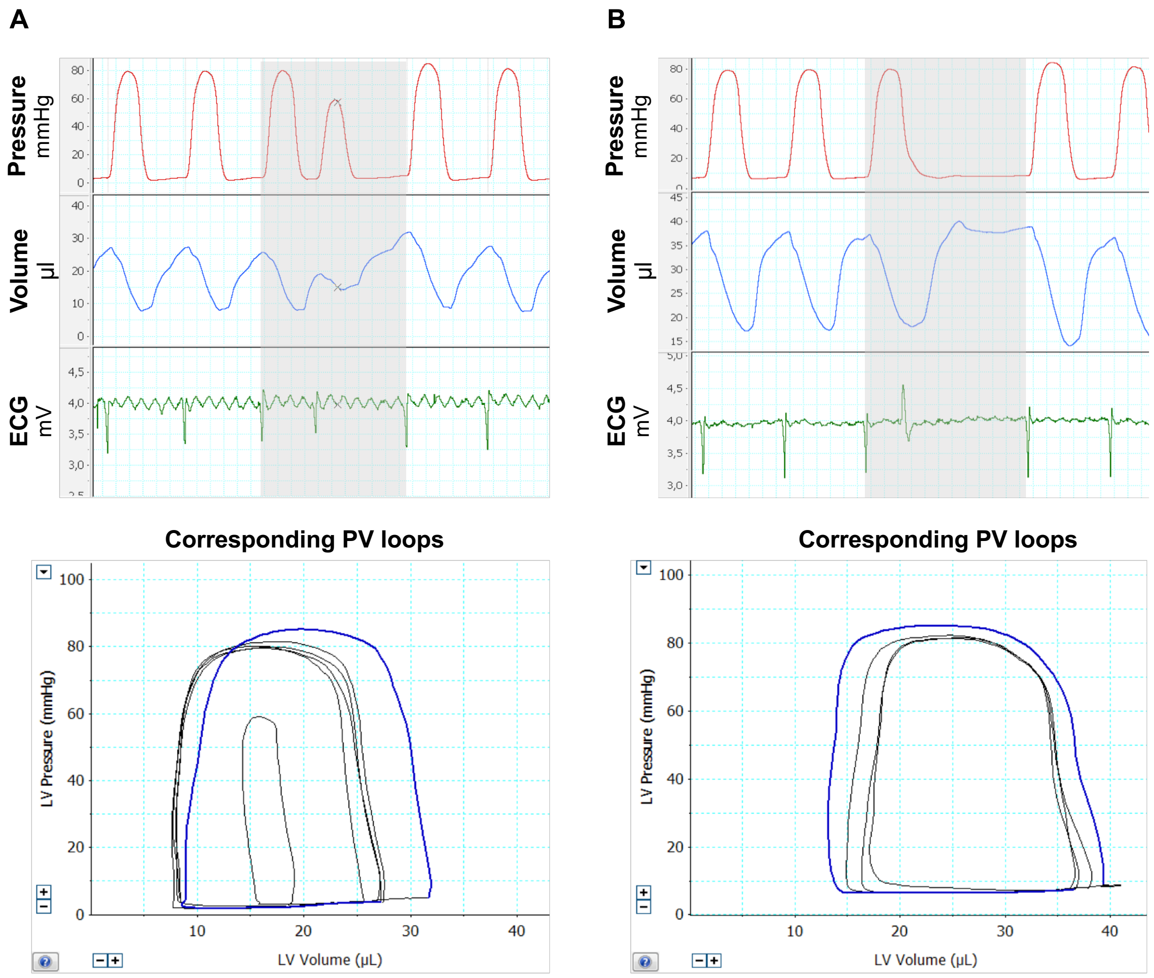

Figure S4. Detection of ectopic beats in pressure-volume recordings. (**A**) Ectopic beat with reduced ejection and (**B**) ectopic beat without ejection. Note that each ectopic beat is followed by a compensatory increased ejection (highlighted blue PV loop), a phenomenon called post-extrasystolic potentiation. Red traces: Intra-ventricular pressure; Blue traces: Intra-ventricular volume; Green traces: Electrocardiogram (ECG). Abbreviations: LV: Left ventricular, PV: pressure-volume.

Supplemental Tables

Table S1: General parameters and calibration settings from mice used to analyse the effect of 2cm H_2O positive end-expiratory pressure.

	No PEEP (n=10)	PEEP (n=8)	p-value
**Body Weight (g)**	33.8 ± 1.1	33.7 ± 2.4	0.9286
**Heart Weight (mg)**	149.5 ± 6.5	145.9 ± 4.8	0.1990
**HW/TL (mg/mm)**	7.87 ± 0.21	7.83 ± 0.24	0.7421
**Age (w)**	20.3 ± 0.1	21.0 ± 0.9	0.0780
**Body Temperature (°C)**	36.9 ± 0.2	36.9 ± 0.2	0.7948
**Gp (RVU)**	19.1 ± 3.3	18.2 ± 1.9	0.4784
**CF (µl/RVU)**	1.87 ± 0.09	1.83 ± 0.16	0.5753

Abbreviations: PEEP: Positive end-expiratory pressure, HW: Heart Weight, TL: Tibia length, Gp: Parallel-Conductance, RVU: Relative Volume Units, CF: Conversion Factor for conductance to volume conversion; n: number of mice. P-values in an unpaired t-test between groups.

Table S2: General parameters and calibration settings from mice used to analyse the effect of early hypertonic saline.

	Late Saline (n=23)	Early Saline (n=15)	p-value
**Body Weight (g)**	30.3 ± 3.4	32.0 ± 3.1	0.1179
**Heart Weight (mg)**	141.5 ± 7.3	142.1 ± 12.5	0.8505
**HW/TL (mg/mm)**	7.69 ± 0.34	7.61 ± 0.6	0.6218
**Age (w)**	18.8 ± 1.8	19.8 ± 0.8	**0.0186**
**Body Temperature (°C)**	37.0 ± 0.2	37.0 ± 0.2	0.7792
**Gp (RVU)**	17.0 ± 2.0	19.6 ± 2.2	**0.0008**
**CF (µl/RVU)**	1.75 ± 0.13	1.77 ± 0.16	0.6813

Abbreviations: Early saline: Hypertonic Saline prior to PV recordings, Late saline: Hypertonic Saline after PV recordings, HW: Heart Weight, TL: Tibia length, Gp: Parallel-Conductance estimated by hypertonic saline injection, RVU: Relative Volume Units, CF: Conversion factor for conductance to volume conversion; n: number of mice. Saline bolus: 10µl bolus of 15% sodium-chloride. Bold digits indicate significant p-values in an unpaired t-test (< 0.05).

Table S3: Extended analysis of pressure-volume parameters from late and early saline groups.

	Systolic Parameters			*Diastolic Parameters*		
**ISO (ng/min)**	**Late saline (n=23)**	**Early saline (n=15)**	**p-value**	**Late saline (n=23)**	**Early saline (n=15)**	**p-value**
	**Heart rate (bpm)**			**End-diastolic Volume (µl)**		
0	476 ± 27	482 ± 33	0.5566	26.8 ± 3.4	37.6 ± 6.9	**< 0.0001**
0.2475	496 ± 27	489 ± 36	0.5222	25.8 ± 3.5	34.2 ± 6.6	**0.0002**
0.825	541 ± 20	517 ± 36	**0.0331**	23.8 ± 3.2	30.1 ± 5.3	**0.0004**
2.475	604 ± 21	580 ± 30	**0.0127**	23 ± 3.1	26.5 ± 5	**0.0257**
8.25	629 ± 19	614 ± 21	**0.0420**	24.5 ± 2.7	25.7 ± 5	0.4179
	**Stroke Volume (µl)**			**End-systolic Volume (µl)**		
0	17.6 ± 2.7	17.2 ± 3.7	0.7163	10.85 ± 4.19	22.99 ± 6.88	**0.0001**
0.2475	18.5 ± 2.3	18.1 ± 3.8	0.6869	8.6 ± 4.1	18.42 ± 6.59	**0.0007**
0.825	20.5 ± 2.7	20.2 ± 4.2	0.7929	4.23 ± 2.5	11.67 ± 5.27	**0.0005**
2.475	22.4 ± 2.1	24.2 ± 4.2	0.1421	1.57 ± 1.8	3.54 ± 5.04	**0.0337**
8.25	24.1 ± 2.3	25.7 ± 4.5	0.2139	1.3 ± 1.52	1.1 ± 4.99	0.6385
	**Cardiac Output (µl/min)**			**End-diastolic Pressure (mmHg)**		
0	8376 ± 1483	8327 ± 2147	0.9386	5.23 ± 0.94	7.63 ± 2.14	**0.0007**
0.2475	9199 ± 1410	8912 ± 2322	0.6712	5.49 ± 0.95	7.23 ± 2.15	**0.0085**
0.825	11097 ± 1639	10533 ± 2693	0.4749	5.49 ± 1.36	6.69 ± 2.11	0.0642
2.475	13515 ± 1366	14075 ± 2843	0.4858	5.25 ± 0.96	6.38 ± 2.03	0.0592
8.25	15137 ± 1564	15817 ± 2984	0.4263	5.52 ± 0.95	6.43 ± 1.95	0.1114
	**Maximum Pressure (mmHg)**			**EDPVR (α)**		
0	81.2 ± 7.0	81.5 ± 9.4	0.9254	1.05 ± 0.79	1.66 ± 1.48	0.1564
0.2475	84.8 ± 7.0	83.7 ± 10.2	0.7293	1.2 ± 0.66	1.81 ± 1.55	0.1662
0.825	86.2 ± 6.0	82.5 ± 8.2	0.1462	1.47 ± 0.74	2.32 ± 1.53	0.0589
2.475	87.8 ± 6.1	84.6 ± 5.7	0.1010	1.92 ± 0.78	2.86 ± 1.56	**0.0444**
8.25	88.7 ± 5.3	86.7 ± 5.5	0.2960	1.82 ± 0.82	3 ± 1.56	**0.0146**
	**PRSW (mmHg)**			**Minimum dP/dt (mmHg/s)**		
0	71.5 ± 8.6	59.5 ± 14.2	**0.0081**	-7936 ± 1260	-7307 ± 2177	0.3224
0.2475	80 ± 12.1	64 ± 20.5	**0.0130**	-8682 ± 1207	-8073 ± 2303	0.3569
0.825	97 ± 14.1	75.5 ± 15.6	**0.0002**	-9050 ± 1169	-8354 ± 1887	0.2154
2.475	110.8 ± 17.1	90.9 ± 14	**0.0004**	-8694 ± 1154	-8693 ± 1215	0.9976
8.25	115.6 ± 14.9	100.5 ± 10.6	**0.0008**	-8679 ± 1284	-8196 ± 1610	0.3379
	**Maximum dP/dt (mmHg/s)**			**Tau (Weiss-Equation)**		
0	7017 ± 1544	6077 ± 2102	0.1495	6.07 ± 0.55	7.1 ± 1.26	**0.0080**
0.2475	8017 ± 1767	6775 ± 2128	0.0716	5.69 ± 0.44	6.46 ± 1.1	**0.0189**
0.825	9480 ± 1850	7625 ± 2154	**0.0108**	5.14 ± 0.51	5.75 ± 0.87	**0.0246**
2.475	12144 ± 1572	10415 ± 2185	**0.0142**	5.01 ± 0.59	5.15 ± 0.72	0.5418
8.25	13658 ± 1160	12458 ± 1394	**0.0103**	5.12 ± 0.67	5.42 ± 0.84	0.2529

Application of 10µl 15% sodium-chloride to correct for parallel-conductance either after (Late saline) or prior to (Early saline) PV-Loop recordings. Abbreviations: ISO: Isoprenaline; PRSW: Preload Recruitable Stroke Work; EDPVR: End-diastolic Pressure-Volume Relationship (alpha coefficient); n: number of mice; p-values between Late saline and Early saline: Bold digits indicate significant p-values in an unpaired t-test (< 0.05).

Table S4: General parameters and calibration settings from mice used to analyse the effects of end-systolic pressure-spikes.

	ESPS (n=6)	No ESPS (n=17)	p-value
**Body Weight (g)**	29.2 ± 3.1	30.6 ± 3.5	0.3896
**Heart Weight (mg)**	142.1 ± 7.2	141.2 ± 7.5	0.8050
**HW/TL (mg/mm)**	7.71 ± 0.31	7.69 ± 0.36	0.9025
**Age (w)**	18.6 ± 1.5	18.8 ± 1.9	0.7475
**Body Temperature (°C)**	37.1 ± 1.5	37.0 ± 0.2	0.7593
**Gp (RVU)**	15.8 ± 1.3	17.4 ± 2.1	0.0511
**CF (µl/RVU)**	1.79 ± 0.14	1.74 ± 0.12	0.4232

Abbreviations: ESPS: End-systolic pressure-spike, HW: Heart weight, TL: Tibia length, Gp: Parallel-Conductance estimated by hypertonic saline injection, RVU: Relative Volume Units, CF: Conversion Factor for conductance to volume conversion; n: number of mice. P-values in an unpaired t-test between groups.

Table S5: Extended analysis of pressure-volume parameters from mice presenting end-systolic pressure-spikes.

ISO (ng/min)	*Systolic Parameters* ESPS (n=6)	No ESPS (n=17)	p-value	*Diastolic Parameters* ESPS (n=6)	No ESPS (n=17)	p-value
	**Heart rate (bpm)**			**End-diastolic Volume (µl)**		
0	469 ± 15	479 ± 31	0.4746	26.9 ± 1.8	26.8 ± 3.9	0.8278
0.2475	489 ± 16	499 ± 30	0.4514	25.7 ± 1.9	25.8 ± 4.0	0.5933
0.825	529 ± 16	545 ± 20	0.0877	23.3 ± 1.3	24.0 ± 3.6	0.5163
2.475	601 ± 27	605 ± 20	0.6962	21.5 ± 1.3	23.5 ± 3.4	0.0601
8.25	631 ± 10	628 ± 21	0.6771	23.0 ± 1.4	25.0 ± 2.9	**0.0407**
	**Stroke Volume (µl)**			**End-systolic Volume (µl)**		
0	16.3 ± 2.9	18.0 ± 2.5	0.2160	12.22 ± 2.16	10.37 ± 3.87	0.3570
0.2475	17.1 ± 2.5	19.0 ± 2.1	0.1019	9.97 ± 2.24	8.12 ± 4.03	0.3660
0.825	19.0 ± 2.1	21.0 ± 2.7	0.0834	5.24 ± 2.23	3.87 ± 3.62	0.2395
2.475	20.6 ± 1.0	23.0 ± 2.1	**0.0015**	1.54 ± 1.69	1.58 ± 3.44	0.9629
8.25	22.4 ± 1.6	24.7 ± 2.2	**0.0192**	1.20 ± 1.17	1.33 ± 2.89	0.8351
	**Cardiac Output (µl/min)**			**End-diastolic Pressure (mmHg)**		
0	7663 ± 1393	8628 ± 1469	0.1744	5.05 ± 0.86	5.30 ± 0.99	0.8622
0.2475	8355 ± 1317	9497 ± 1352	0.0898	5.24 ± 1.22	5.58 ± 0.86	0.5928
0.825	10057 ± 1263	11464 ± 1627	0.0524	5.20 ± 1.44	5.60 ± 1.36	0.5716
2.475	12391 ± 885	13911 ± 1297	**0.0073**	5.18 ± 1.46	5.27 ± 0.78	0.8858
8.25	14114 ± 1045	15498 ± 1578	**0.0305**	5.35 ± 1.45	5.58 ± 0.75	0.7168
	**Maximum Pressure (mmHg)**			**EDPVR (α)**		
0	79.3 ± 6.6	81.9 ± 7.2	0.4255	0.71 ± 0.31	1.17 ± 0.87	0.1616
0.2475	81.1 ± 6.1	86.1 ± 7.0	0.1075	0.95 ± 0.40	1.28 ± 0.72	0.2589
0.825	83.1 ± 5.7	87.3 ± 5.9	0.1561	1.26 ± 0.62	1.54 ± 0.78	0.3961
2.475	90.0* ± 7.3	88.6 ± 6.3	0.7453	1.91 ± 0.98	1.93 ± 0.73	0.9623
8.25	99.8* ± 8.8	89.5 ± 5.6	**0.0347**	2.04 ± 1.01	1.75 ± 0.76	0.5406
	**PRSW (mmHg)**			**Minimum dP/dt (mmHg/s)**		
0	76.2 ± 8.3	69.8 ± 8.3	0.0897	-7606 ± 1013	-8053 ± 1345	0.3563
0.2475	79.6 ± 8.6	80.1 ± 13.3	0.9710	-7885 ± 796	-8964 ± 1218	**0.0221**
0.825	97.0 ± 11.3	97.0 ± 15.3	0.9929	-8181 ± 834	-9357 ± 1130	**0.0197**
2.475	107.3 ± 11.8	112.0 ± 18.8	0.4861	-8928 ± 696	-8612 ± 1286	0.4650
8.25	116.4 ± 9.7	115.4 ± 16.7	0.8533	-9807 ± 1410	-8281 ± 1001	**0.0453**
	**Maximum dP/dt (mmHg/s)**			**Tau (Weiss-Equation)**		
0	6541 ± 1320	7185 ± 1618	0.3275	6.18 ± 0.46	6.03 ± 0.58	0.3783
0.2475	7160 ± 1458	8319 ± 1804	0.1209	5.92 ± 0.56	5.61 ± 0.39	0.2306
0.825	8522 ± 1868	9817 ± 1774	0.1756	5.39 ± 0.74	5.05 ± 0.40	0.3225
2.475	11517 ± 1515	12365 ± 1575	0.2727	5.26 ± 0.52	4.93 ± 0.60	0.2182
8.25	13394 ± 831	13751 ± 1264	0.4485	5.52 ± 0.45	4.97 ± 0.69	**0.0445**

Abbreviations: ISO: Isoprenaline; PRSW: Preload Recruitable Stroke Work; EDPVR: End-diastolic Pressure-Volume Relationship (alpha coefficient); ESPS: end-systolic pressure-spike; n: number of mice; p-values in an unpaired t-test between recordings with and without ESPS: Bold digits indicate significant p-values (< 0.05). *Before manual correction as described in Figure 5.
